# Agitation in cognitive disorders: International Psychogeriatric Association provisional consensus clinical and research definition

**DOI:** 10.1017/S1041610214001963

**Published:** 2014-10-14

**Authors:** Jeffrey Cummings, Jacobo Mintzer, Henry Brodaty, Mary Sano, Sube Banerjee, D.P. Devanand, Serge Gauthier, Robert Howard, Krista Lanctôt, Constantine G. Lyketsos, Elaine Peskind, Anton P. Porsteinsson, Edgardo Reich, Cristina Sampaio, David Steffens, Marc Wortmann, Kate Zhong

**Affiliations:** 1 Cleveland Clinic Lou Ruvo Center for Brain Health, Las Vegas, Nevada, USA; 2 Roper St. Francis Hospital, The Clinical Biotechnology Research Institute, South Carolina, USA; 3 Centre for Health Brain Ageing, The University of New South Wales, Sydney, New South Wales, Australia; 4 Mount Sinai School of Medicine, New York, New York, USA; 5 Brighton and Sussex Medical School, Trafford Centre for Medical Research, University of Sussex, Brighton, East Sussex, UK; 6 New York State Psychiatric Institute, Riverside Drive, New York, New York, USA; 7 McGill Center for Studies in Aging, Douglas Mental Health University Institute Montreal, Montreal, Quebec, Canada; 8 Institute of Psychiatry, King's College of London, London, UK; 9 Sunnybrook Research Institute, University of Toronto, Toronto, Ontario, Canada; 10 The Johns Hopkins Memory and Alzheimer's Treatment Center, The Johns Hopkins Bayview Medical Center, Baltimore, Maryland, USA; 11 VA Puget Sound Health Care System, Columbian Way, Seattle, Washington, USA; 12 Monroe Community Hospital, Rochester, New York, USA; 13 Department of Neuroscience, Hospital Julio Mendez, University of Buenos Aires, Buenos Aires, Argentina; 14 CHDI Foundation, Princeton, New Jersey, USA; 15 Department of Psychiatry, University of Connecticut Health Center, Farmington, Connecticut, USA; 16 Alzheimer's Disease International, London, UK; 17 Cleveland Clinic Lou Ruvo Center for Brain Health, Las Vegas, Nevada, USA

**Keywords:** Alzheimer’s disease, agitation, cognitive impairment, aggression, International Psychogeriatric Association, Food and Drug Administration, clinical trials, intervention, epidemiology

## Abstract

**Background:**

Agitation is common across neuropsychiatric disorders and contributes to disability, institutionalization, and diminished quality of life for patients and their caregivers. There is no consensus definition of agitation and no widespread agreement on what elements should be included in the syndrome. The International Psychogeriatric Association formed an Agitation Definition Work Group (ADWG) to develop a provisional consensus definition of agitation in patients with cognitive disorders that can be applied in epidemiologic, non-interventional clinical, pharmacologic, non-pharmacologic interventional, and neurobiological studies. A consensus definition will facilitate communication and cross-study comparison and may have regulatory applications in drug development programs.

**Methods:**

The ADWG developed a transparent process using a combination of electronic, face-to-face, and survey-based strategies to develop a consensus based on agreement of a majority of participants. Nine-hundred twenty-eight respondents participated in the different phases of the process.

**Results:**

Agitation was defined broadly as: (1) occurring in patients with a cognitive impairment or dementia syndrome; (2) exhibiting behavior consistent with emotional distress; (3) manifesting excessive motor activity, verbal aggression, or physical aggression; and (4) evidencing behaviors that cause excess disability and are not solely attributable to another disorder (psychiatric, medical, or substance-related). A majority of the respondents rated all surveyed elements of the definition as “strongly agree” or “somewhat agree” (68–88% across elements). A majority of the respondents agreed that the definition is appropriate for clinical and research applications.

**Conclusions:**

A provisional consensus definition of agitation has been developed. This definition can be used to advance interventional and non-interventional research of agitation in patients with cognitive impairment.

## Introduction

Agitation is a common clinical manifestation of many neuropsychiatric disorders. It is a frequent manifestation of Alzheimer's disease (AD), frontotemporal dementia (FTD), dementia with Lewy bodies (DLB), and other dementia syndromes (Ballard and Corbett, 2010; Manoochehri and Huey, 2012; Bruns and Josephs, 2013). It occurs in schizophrenia, bipolar illness, and depression (Gonzalez *et al*., 2013; Swann, 2013). While agitation may include aggressive behaviors, it is not identical to aggression, and agitation can occur without aggression (e.g. pacing, rocking, repetitious mannerisms). Agitation can precipitate institutionalization (Okura *et al*., 2011), diminishes the quality of life of patients and caregivers (Khoo *et al*., 2013), and, when severe, may require treatment with medications (Herrmann and Lanctôt, 2007). There is an emerging biology of agitation, and frontal lobe dysfunction is implicated in both clinical and neuroimaging studies (Senanarong *et al*. 2004; Bruen *et al*., 2008). Treatment of agitation – both pharmacologic and non-pharmacologic – is an unmet need in the care of patients with cognitive impairment (Herrmann and Lanctôt, 2007; Gitlin *et al*., 2012).

In spite of the framework of studies that have begun to increase understanding of agitation, there is no commonly accepted consensus description of this common clinical phenomenon (Laughren, 2001). Lay definitions of agitation are non-specific and include states of excitement, disturbance, or worry. A consensus definition of agitation applicable in the setting of cognitive impairment would facilitate a wide spectrum of research, including pharmacologic and non-pharmacologic intervention studies, epidemiologic investigations of agitation, clinical studies, and research on the neurobiology of this behavior. A definition would also provide a common framework for diagnostic nomenclatures such as the International Classification of Diseases and Related Health Problems (ICD; (World Health Organization, 2014) and the Diagnostic and Statistical Manual of Mental Disorders (DSM; (American Psychiatric Association, 2013). In addition, clinically relevant definitions have important regulatory applications; when agents possibly appropriate for the treatment of agitation are presented to the Food and Drug Administration (FDA), European Medicines Agency (EMA), or other licensing authorities, the treatment indication must be defined using language useful to clinicians caring for patients with the condition. Without a consensus definition, it is difficult to compare studies or to know what range of behaviors were included in a study of “agitation.”

Rating scales such as the Cohen-Mansfield Agitation Inventory (Cohen-Mansfield *et al*., 1989), Neuropsychiatric Inventory (NPI; Cummings *et al.*, 1994), or Behavioral Pathology in Alzheimer's Disease (BEHAVE-AD; Reisberg *et al*., 1987; De Deyn and Wirshing, 2001) are often used to identify patients for clinical trials of anti-agitation agents and to measure the clinical symptoms in other descriptive and intervention studies. Rating scales, however, are not definitions; rather, they are means of measuring the frequency or severity of symptoms. Most clinicians do not use rating scales for routine care of patients. To assist in defining populations for clinical care and research, a definition that is not dependent on a particular rating scale is needed.

The International Psychogeriatric Association (IPA) has an established leadership role in the field of geriatric behavioral health, including agitation, and has led initiatives involved with this topic (Finkel *et al*., 1996; Reisberg *et al*., 1997; Draper, 1999; Finkel, 2000). To advance the study of agitation by establishing a consensus definition, the IPA formed an Agitation Definition Working Group (ADWG) to produce a provisional consensus definition of agitation. The ADWG conducted a broadly inclusive process, involving the IPA and its affiliate members, employing electronic means of participant engagement, holding a face-to-face meeting with international representation, and using survey-based methods. The ADWG was made up of the IPA leadership and other stakeholders interested in the neuropsychiatric aspects of AD and other disorders. The ADWG implemented a transparent process that included nearly 1,000 survey respondents and engaged the memberships of the IPA, IPA affiliates, and other organizations involved in the care and research of neuropsychiatric disorders in patients with cognitive impairment. Here the process is described, the definition is presented, and the elements of the definition are discussed.

## Methods

### Consensus-building process

#### Initial formulation

The literature was reviewed (by Jeffrey Cummings), past definitions of agitation identified, and common elements of the definitions reviewed. A preliminary survey of these definitions and elements to be considered for inclusion was developed and presented to the IPA leadership.

#### Survey 1

The ADWG was formed to guide this project. The ADWG reviewed the proposed survey, adding or deleting elements to enhance the focus and clarity of the questions. The revised survey was sent electronically to the IPA membership and affiliate members (organizations that share the mission of advancing clinical practice, research, and education to improve the mental health of the elderly). The survey collected demographic information on the respondents, determined the preferences among five definitions of agitation used in the literature (Kong, 2005), asked if the definition should be limited to cognitive impairment syndromes, established key behavioral elements to be included in a definition, and interrogated whether the respondents considered agitation and anxiety to be the same and whether agitation and aggression are the same.

#### International expert consensus meeting

Following completion and analysis of the first survey, an international expert consensus meeting was held to develop a draft definition based on a review of the literature and information derived from the survey. The members of the ADWG present at the meeting include the authors of this paper. In this meeting a draft definition was developed that represented a consensus of those present. Following the expert meeting, a preliminary draft of the definition was developed and circulated to all members of the ADWG. Through multiple electronic exchanges a final consensus was reached (Table 1). Not all aspects of the consensus definition were unanimously endorsed by all participants but a majority of stakeholders agreed on the elements.

**Table 1.** Consensus provisional definition of agitation in cognitive disorders

A. The patient meets criteria for a cognitive impairment or dementia syndrome (e.g. AD, FTD, DLB, vascular dementia, other dementias, a pre-dementia cognitive impairment syndrome such as mild cognitive impairment or other cognitive disorder).	
B. The patient exhibits at least one of the following behaviors that are associated with observed or inferred evidence of emotional distress (e.g. rapid changes in mood, irritability, outbursts). The behavior has been persistent or frequently recurrent for a minimum of two weeks’ and represents a change from the patient's usual behavior.	
(a) Excessive motor activity (examples include: pacing, rocking, gesturing, pointing fingers, restlessness, performing repetitious mannerisms).	
(b) Verbal aggression (e.g. yelling, speaking in an excessively loud voice, using profanity, screaming, shouting).	
(c) Physical aggression (e.g. grabbing, shoving, pushing, resisting, hitting others, kicking objects or people, scratching, biting, throwing objects, hitting self, slamming doors, tearing things, and destroying property).	
C. Behaviors are severe enough to produce excess disability, which in the clinician's opinion is beyond that due to the cognitive impairment and including at least one of the following:	
(a) Significant impairment in interpersonal relationships.	
(b) Significant impairment in other aspects of social functioning.	
(c) Significant impairment in ability to perform or participate in daily living activities.	
D. While co-morbid conditions may be present, the agitation is not attributable solely to another psychiatric disorder, suboptimal care conditions, medical condition, or the physiological effects of a substance.	

To develop further consensus beyond the ADWG, other key stakeholder groups received the provisional definition for comments, including the members of the Neuropsychiatric Syndrome Professional Interest Area of ISTAART (Geda *et al*., 2013) comprising thought leaders in the area of neuropsychiatric aspects of AD.

#### Survey 2

Once an expert consensus from the ADWG was achieved, the IPA and affiliate members were re-contacted to provide additional input into the definition and its acceptability for clinical and research studies, including prospective validation investigations. Six thousand emails were sent; there were 350 respondents (7%) in this phase of the consensus development process.

#### Report preparation

Once the second survey was complete, this report containing a description of the consensus process, the definition, and the elements of the definition was prepared (Jeffrey Cummings) and circulated among the ADWG members. Agreement was reached on the text describing the provisional definition and the manuscript was submitted to *International Psychogeriatrics* for review.

## Results

### Survey 1

Six thousand emails were sent; 557 individuals responded. The response rate from IPA members was 30.1%. Many members of the IPA and affiliate organizations are involved predominantly with mood disorders, psychosis, or other non-dementia aspects of geriatric psychiatry. Of the 557 respondents, 382 were physicians, including 292 psychiatrists. Other professional disciplines providing responses included nurses (57), psychologists (50), occupational therapists (13), social workers (14), and others/no responses (41). Three hundred eighty-eight (70%) of the respondents had been in practice for more than ten years.

This initial survey provided valuable insights from those involved in the care of agitated patients, and key elements of the definition were identified. Table 2 shows the responses to five existing definitions of agitation (Kong, 2005). Three of the definitions had relatively high acceptability (22–34% rating as the “best definition”).

**Table 2.** Five proposed definitions of agitation in dementia and the number of respondents who ranked each of them as the best or the second-best definition

definition	percentage of respondents who ranked the definition as the best	percentage of respondents who ranked the definition as the second best
The patient has periods when he refuses to cooperate, won't let people help, or is hard to handle.	4	4
Excessive motor activity associated with a feeling of inner tension. The activity is usually non-productive and repetitious and consists of such behavior as inability to sit still, pacing, wringing of hands, and pulling at clothes.	31	19
Inappropriate verbal, vocal, or motor activity that is not explained by needs or confusion *per se*. It includes behaviors such as aimless wandering, pacing, cursing, screaming, biting, and fighting.	22	38
Vocal or motor behavior that is either disruptive, unsafe, or interferes with the delivery of care in a particular environment. It included four behavioral areas such as vocalization, motor disturbances, aggressiveness, and resisting care.	34	26
Those observed patient behaviors that communicate to others that the patient is experiencing an unpleasant state of excitement and which remain after interventions to reduce internal or external stimuli by managing resistiveness, alleviating aversive physical signs, and decreasing sources of accumulated stress have been carried out.	9	12

The ADWG surveyed essential elements of the definition of agitation. The percentage of respondents identifying specific adjectives as key behavioral elements of the definition included: excessive (71%), inappropriate (54%), repetitive (46%), observable (64%), dangerous (24%), and disruptive (56%) (Table 3).

**Table 3.** Proposed elements of a definition of agitation and the percentage of respondents endorsing that item as an essential element of a definition of agitation

element	percentage of respondents endorsing the elements as a key component of a definition of agitation
Excessive	71
Inappropriate	54
Repetitive	46
Observable	64
Dangerous	24
Disruptive	56

Of the items listed as possible behaviors to be included in a definition of agitation, the following were endorsed by at least 50% of the respondents: pacing, aimless wandering, verbal aggression, constant unwarranted requests for attention or help, hitting others, hitting self, pushing people, throwing things, general restlessness, screaming, resistiveness, hurting self, hurting others, tearing things or destroying property, shouting, and kicking furniture (Table 4). This information guided the elements included in the definition by the ADWG.

**Table 4.** Behaviors that survey respondents indicated should be included in a definition of agitation

item	percentage of respondents who thought the item belonged to the definition of agitation
Pacing	68
Aimless wandering	52
Spitting at meals	29
Spitting at people	40
Cursing	42
Verbal aggression	71
Constant unwarranted requests for attention or help	53
Repetitive questions	35
Repetitive sentences	31
Hitting others	58
Hitting self	57
Grabbing people	48
Pushing people	54
Throwing things	56
General restlessness	80
Screaming	63
Biting	49
Scratching	48
Trying to get to a different place (e.g. out of the room or building)	48
Intentional falling	16
Complaining	20
Negativism	21
Resistiveness	54
Eating/drinking inappropriate substances	15
Hurting self	54
Hurting others	53
Handling things inappropriately	26
Hiding things	11
Hoarding things	12
Tearing things or destroying property	62
Performing repetitious mannerisms	45
Making verbal sexual advances	23
Making physical sexual advances	24
Making strange noises (weird laugher or crying)	38
Stubbornness	17
Shouting	62
Slamming doors intentionally	46
Kicking furniture	52

When queried as to whether agitation and anxiety were the same, overlapping, or distinct concepts, most of the respondents found them to be overlapping (0.3%, 61%, and 37% respectively). When asked whether agitation and aggression are the same, overlapping, or distinct, 0.8%, 66%, and 32% endorsed each option.

### Survey 2

Table 5 lists the questions of the survey regarding the definition developed by the ADWG and the responses of the participants. All elements of the definition surveyed were rated as “strongly agree” or “somewhat agree” by a majority of the respondents (ranging from 68.2% for “the exclusion criterion is clear” to 88.8% for “the physical aggression components are captured appropriately in the definition”). A majority agreed (strongly or somewhat) that the definition is appropriate for research application. For non-interventional descriptive clinical research, 44% strongly agreed and 33.7% somewhat agreed with the appropriateness of the definition (77.7% agreed); for pharmacologic interventional clinical research, 43.7% strongly agreed and 31.7% somewhat agreed (75.4% agreed); and for clinical trials 39.2% and 36% agreed strongly and somewhat respectively (75.2% agreed).

**Table 5.** Questions of survey 2 exploring the acceptability of the draft definition and the percentage of participants who responded as strongly agree or somewhat agree

survey question	percentage of respondents who strongly agree	percentage of respondents who somewhat agree
The definition captures what I mean when I use the term “agitation” to describe one of my patients.	50.8	35.1
The inclusion criteria – A, B, C – are sufficiently clear for clinical application.	51.7	31.7
The inclusion criteria – A, B, C – are sufficiently clear for research application.	35.7	37.7
The exclusion criterion – D – is sufficiently clear for clinical application.	38.5	36.8
The exclusion criterion – D – is sufficiently clear for research application.	26.0	42.2
The subjective aspects of the syndrome are captured appropriately – observed or inferred evidence of emotional distress (e.g. rapid changes in mood, irritability, outbursts). The behavior has been sustained or persistent for a minimum of two weeks in duration and represents a change from the person's usual behavior.	42.8	35.4
The physical aggression aspects of the syndrome are captured appropriately – grabbing, shoving, pushing, resisting, hitting others, kicking objects or people, scratching, biting, throwing objects, hitting self, slamming doors, tearing things, and destroying property.	65.7	23.1
The verbal aspects of the syndrome are captured appropriately – yelling, speaking in an excessively loud voice, using profanity, screaming, shouting.	59.7	28.2
The definition is appropriate as a means of identifying patients for non-interventional descriptive clinical research.	44.0	33.7
The definition is appropriate as a means of identifying patients for non-pharmacologic interventional clinical research.	43.7	31.7
The definition is appropriate as a means of identifying patients for clinical trials.	36.2	36
The definition is appropriate as a foundation for validation studies of its sensitivity and specificity.	39.4	36.2

When queried whether the definition should be limited to cognitive impairment syndromes – the approach taken by the ADWG – 67.4% said “yes.”

## Discussion

### Elements of the consensus definition

The provisional consensus definition uses the DSM style for defining a disorder as one that produces disability (American Psychiatric Association, 2013). The definition is limited to patients with cognitive impairment, requires evidence of emotional distress, requires one of the three observable types of behavior (excessive motor activity, verbal aggression, or physical aggression), specifies that the behavior causes excess disability, and notes that the behaviors cannot be solely attributable to a suboptimal care environment or another disorder such as psychiatric illness, medical illness, or effects of a substance.

#### Provisional consensus definition

The definition is labeled as “provisional” because it may evolve as it is subjected to prospective validation. It represents an important starting point in a dynamic dialogue that will evolve with clinical application, research, and review. Validation studies are expected to show that some elements are better suited to clinical and research applications than others.

The definition was labeled as a “consensus” because a majority of stakeholders involved in the process concurred with the current definition. Not all elements were unanimously endorsed; a consensus was achieved on all aspects of the definition.

#### Limitation to syndromes with cognitive impairment

Agitation occurs in many disorders and is not limited to conditions with cognitive impairment (Ballard and Corbett, 2010; Manoochehri and Huey, 2012; Bruns and Josephs, 2013; Gonzalez *et al.*, 2013; Swann, 2013). The ADWG and the participating members of the surveys favored limiting the definition to disorders with cognitive impairment. Sixty-seven percent of Survey 2 respondents agreed with this approach.

There are currently separate definitions for psychosis of AD and psychosis in schizophrenia even though they have shared elements such as delusions and hallucinations (Jeste and Finkel, 2000); similarly there are definitions of major depression and depression in AD that are overlapping but not identical (Olin *et al.*, 2003). Similar considerations may apply to agitation, and we chose to develop a definition explicitly for patients with cognitive impairment. Further research may lead to modifications of this approach.

The FDA has expressed concern about “pseudo-specificity” of syndromes that are artificially assigned to one disorder when they represent general syndromes for which drugs are already approved (Cummings and Jeste, 2007), and the ADWG wished to avoid constructing a definition that might raise this issue. Our definition applies specifically to the type of agitation observed in patients with cognitive impairment based on the unique relationships of agitation with aspects of cognition (Senanarong *et al*., 2004); a distinct pathophysiology (Bruen *et al.*, 2008; Trzepacz *et al.*, 2013); and possibly a differential response to treatment compared with syndromes such as psychosis of AD (Schneider *et al.*, 2006).

#### Subjective aspect

The definition requires that there be observed or inferred evidence of emotional distress. Examples of behaviors indicative of emotional distress are provided, including rapid changes in mood, irritability, or emotional outbursts. The ADWG required this subjective element because agitation as conceived by the panel includes the concept that the patient is upset or distressed, and the agitated behavior is an expression of this emotional state. A person could exhibit purposeful aggression with behaviors overlapping with the definition but would not be classified as “agitated” if there was no associated distress. It is also important that patients not be treated when their behavior is upsetting to someone else (a family member or caregiver) but not to the patient. Requiring the subjective element of the definition makes this less likely. It is not possible to directly observe emotional states, and they must be inferred from observable behaviors. In all, 78.2% of the Survey 2 respondents strongly (42.8%) or somewhat (35.4%) agreed that this aspect of agitation was captured appropriately by the proposed definition.

#### Sustained

A single episode or short-lived period of behavioral change would not meet the definition for agitation developed by the ADWG. The definition applies to a chronic or at least long-lasting state that has been sustained, persisted, or been frequently recurrent for at least two weeks. This aspect of the definition will assist with intervention studies (pharmacologic and non-pharmacologic) where interventional groups are compared and sustained behavioral changes are required to see a difference in the study outcomes.

#### Change from previous behavior

The ADWG limited the definition of agitation to patients with cognitive impairment. Behaviors that preceded the onset of the associated cognitive disorder would not be included in this definition. This aspect of the consensus definition is similar to the precedent adapted in the definition of psychosis of AD (Jeste and Finkel, 2000).

#### Observable behaviors

The patient must have at least one type of observable agitated behavior. The behaviors can include *excessive motor activity* (such as pacing, rocking, gesturing, pointing fingers, restlessness, performing repetitious mannerisms), *verbal aggression* (e.g. yelling, speaking in an excessively loud voice, using profanity, screaming, shouting), or *physical aggression* (e.g. grabbing, shoving, pushing, resisting, hitting others, kicking objects or people, scratching, biting, throwing objects, hitting self, slamming doors, tearing things, and destroying property). These behaviors are the core aspects of the agitation syndrome; they reflect the behaviors endorsed in Survey 1 as comprising agitation. The ADWG recognized that agitated patients exhibit different repertoires of behaviors and that, in the presence of emotional distress, any of the behaviors described would fulfill this criterion of the definition. Clinician judgment is not prohibited by the definition and behaviors other than those listed may be present in agitated patients. In all, 88.8% of the Survey 2 respondents strongly (65.7%) or somewhat (23.1%) agreed that the proposed definition appropriately captured the physical aggression aspects of agitation, and 87.9% strongly (59.7%) or somewhat (28.2%) agreed regarding the verbal aspects of agitation.

#### Excess disability

A disorder is defined as producing disability (American Psychiatric Association, 2013), and the ADWG incorporated this aspect of the definition as a measure of the clinical meaningfulness of the behavior. If the behavior produces no disability for the individual in terms of interpersonal relationships, social function, or impact on daily living activities, then it would not meet this criterion of the definition. The disability must be more than can be attributed solely to the cognitive impairment syndrome. Clinician judgment will be required to make this determination.

#### Necessary exclusions

Agitation has many possible causes and is seen across a broad range of neuropsychiatric illnesses (Ballard and Corbett, 2010; Manoochehri and Huey, 2012; Bruns and Josephs, 2013; Gonzalez *et al*., 2013; Swann, 2013). The definition proffered by the ADWG requires the presence of an underlying cognitive impairment syndrome such as AD, FTD, DLB, or a prodromal dementia state. In some cases, patients with these disorders may have other psychiatric illness, medical illness, use substances, or be in suboptimal care environments that can cause agitated behaviors. The ADWG definition requires that the agitation not be attributable solely to one of these co-existing conditions. Clinician judgment may be required to make this determination. The exclusion aspects of the definition had some of the lowest endorsements on Survey 2 as 38.5% strongly agreed and 36.8% somewhat agreed that the exclusions were adequate for clinical application; 26% strongly agreed and 42.2% somewhat agreed that the exclusions were adequate for research application.

### Comment on the definition

The ADWG definition of agitation with cognitive impairment is broad and assumes that agitation is a syndrome and not a response to another disorder. An alternative approach is to define an “agitation with psychosis,” “agitation with depression,” etc. An extensive literature establishes that agitation can be seen in the absence of concomitant psychopathology; neuroimaging studies suggest unique regional dysfunction that is distinct from other disorders (Bruen *et al.*, 2008; Trzepacz *et al.*, 2013); and clinical trials demonstrate that the pharmacologic response of agitation may differ from that of other types of behavioral disturbances (Schneider *et al.*, 2006). Therapies have been approved for agitation in multiple psychiatric settings (e.g. aripiprazole for acute treatment of agitation with schizophrenia or bipolar I disorder), setting a precedent for viewing agitation as a distinct syndrome. While no drug has been approved for agitation *per se* (i.e. without aggression, depression, or psychosis), several psychosocial interventions have shown benefit in reducing agitated behavior (Low *et al.*, 2013). The ADWG favored defining agitation as a distinct clinically identifiable syndrome.

Using a syndromal definition of clinical phenomena within neurologic disorders has a precedent in the identification of pseudo-bulbar affect (PBA) and the approval of dextromethorphan/quinidine for PBA across neurologic disorders after having demonstrated efficacy in amyotrophic lateral sclerosis and multiple sclerosis. A similar strategy of showing efficacy for anti-agitation agents across several disorders could be adopted in anti-agitation drug development programs.

The relationship of agitation to aggression sparked discussion. These are overlapping but not identical concepts (Cohen-Mansfield and Mintzer, 2005). Agitation can occur in the absence of aggression, and predatory aggression can occur without agitation; it is unlikely that aggression occurs without agitation in the cognitive impairment syndromes described by the ADWG. Sixty-six percent of the survey respondents consider agitation and aggression as overlapping concepts; 32% consider them to be distinct. Aggression may be a more severe form of agitation or it may occur in differing biological or psychological circumstances. The comparative response profiles to pharmacologic or non-pharmacologic interventions of agitation with and without aggression have not been comprehensively explicated. There is currently insufficient evidence to conclude that agents or interventions used for aggressive agitation would necessarily work or be warranted in non-aggressive agitation. The ADWG definition criterion B allows both agitation without aggression (excessive motor activity) and with aggression (verbal aggression and physical aggression). Investigators using this definition of agitation should note which elements of criterion B were met (e.g. excessive motor activity, verbal aggression, or physical aggression). Longitudinal comparison of studies will help define the relationship between agitation and aggression, and the ADWG definition will advance understanding of this aspect of agitation.

This definition will be useful in the study of psychosocial interventions and in clinical trials of anti-agitation agents. The absence of a consensus definition may have contributed to the lack of activity in developing new therapies for agitation with cognitive impairment; no agent has been approved for treatment of this syndrome in spite of extensive documentation of the disability associated with agitation. Study entry would require that the participants have agitation as defined by the ADWG. A minimum baseline severity level on an agitation scale would typically be required. An outcome measure – usually an agitation measure different from the one used to define entry severity – would be used to compare baseline with end-of-study scores for the active treatment and the placebo (or active comparator) control group. It may be useful to control for other behavioral features such as mood changes or psychosis. The ADWG definition could play a critical role in defining the patient population for such trials.

The development of a provisional definition of agitation is the first step in advancing a research agenda for the definition. Validity studies using other agitation assessments, reliability of the application of the definition, usefulness in clinical trials, usefulness in non-pharmacologic research, and real-world application in clinical and healthcare settings will lead to refinements and adjustments that will enhance the definition and advance the study of neuropsychiatric syndromes in cognitive impairment disorders.

## Conflict of interest declaration

The sponsors, Otsuka and Avanir Pharmaceutricals, gave unrestricted education grants to help support the international meeting that was part of the definition-building process.

Dr. Jeffrey Cummings has provided consultation to Acadia, ADAMAS, Anavex, Avanir, Boehinger-Ingelheim, Eisai, EnVivo, GE Healthcare, Genentech, Lilly, Lundbeck, Medavante, Merck, Novartis, Otsuka, Pfizer, Prana, QR Pharma, Resverlogix, Roche, Sonexa, Suven, Takeda, and Toyoma companies. Dr. Cummings owns the copyright of the Neuropsychiatric Inventory.

Professor Sube Banerjee has received consultancy fees, honoraria for speaking, and support in attending conferences from the makers of anti-dementia medication and other psychotropics, including Abbvie, Eisai, Pfizer, Novartis, Lilly, and Shire.

Professor Henry Brodaty has served as investigator for Janssen, Lilly, Medivation, Merck, Sanofi, Servier, and Tau Therapeutics, and has been on the advisory boards of Pfizer, Novartis, Janssen, Lundbeck, and Nutricia. Dr. Brodaty has provided consultation to Baxter, Lilly, Merck, and Nutricia.

D.P. Devanand has received research support from Eli Lilly, and has provided consultation to AbbVie.

Dr. Serge Gauthier has been on the scientific advisory board or investigator with Abbvie, Affiris, Eisai, Lilly, Navidea, Novartis, Pfizer, Sanofi-Synthelabo, Servier, and TauRx.

Professor Robert Howard reports that he has no relevant disclosures to make.

Dr. Krista Lanctôt has had research support and/or speaker's honoraria from Abbvie Canada, Lundbeck Canada Inc., Pfizer Canada Inc., Janssen Ortho, and Rocheand Wyeth.

Dr. Constantine Lyketsos has received grant support (research or CME) from Associated Jewish Federation of Baltimore, Weinberg Foundation, Forest, GlaxoSmithKline, Eisai, Pfizer, Astra-Zeneca, Lilly, Ortho-McNeil, Bristol-Myers, Novartis, National Football League, Elan, and Functional Neuromodulation. Dr. Lyketsos has been a consultant or advisor to Astra-Zeneca, GlaxoSmithKline, Eisai, Novartis, Forest, Supernus, Adlyfe, Takeda, Wyeth, Lundbeck, Merz, Lilly, Pfizer, Genentech, Elan, NFL Players Association, NFL Benefits Office, Avanir, Zinfandel, BMS, Abvie, Janssen, and Orion. Honorarium or travel support has been received from Pfizer, Forest, GlaxoSmithKline, and Health Monitor.

Dr. Jacobo Mintzer reports receipt of grants to his institution from Takeda Global Research and Development Center, Pfizer, Genentech, Merck, Accera, Elan, Avanir, F. Hoffmann-La Roche, Novartis, Baxter, Eli Lilly, Wyeth, and Janssen Alzheimer Initiative; and employment with NeuroQuest, and BioPharma Connex.

Dr. Elaine Peskind has been on the speakers’ bureau for Forest Pharmaceuticals and on the Advisory Committee for Avanir. Dr. Peskind served on the Adjudication Committee for Takeda Pharmaceuticals.

Dr. Anton Porsteinsson has received a grant for his institution from Avanir, Eisai, Elan, Genentech/Roche, Janssen Alzheimer Initiative, Merck, Pfizer, and Toyama. He has provided consultation for Elan, Janssen Alzheimer Initiative, and TransTech Pharma. Dr. Porsteinsson has membership on data safety and monitoring boards for Quintiles.

Dr. Edgardo Reich has participated as investigator in different pharmaceutical-sponsored trials for Ely Lilly, Glaxo, Forest, Mertz. Astra Zeneca, Novartis, Roche, and Janssen. He has received travel grants from Bayer, MerckSerono, Novartis, Mertz, and Teva.

Dr. Cristina Sampaio has been a consultant for Abbvie, Alkermes, AstraZeneca, Avanir, Biogen, BMS, Chelsea, Genzyme, Lilly, Lundbeck, Otsuka, Pfizer, Roche, Sanofi, Servier, Takeda, and Teva.

Dr. Mary Sano has been on the Scientific Advisory Board for Medication and as a consultant for Bayer Schering Pharma, Bristol-Meyers Squibb, Elan, Genentech, Medivation, Medpace, Pfizer, Janssen, Takeda, and United Biosource.

Dr. David Steffens is supported through grants from the National Institute of Mental Health, and he receives textbook royalties from the American Psychiatric Press.

Dr. Marc Wortmann reports no other salaries than from Alzheimer's Disease International.

Dr. Kate Zhong has provided consultation to Avanir, Pfizer, Janssen, and Wyeth Pharmaceuticals.

## Description of authors’ roles

J. Cummings, J. Mintzer, H. Brodaty, and M. Sano formulated the research questions, designed the study, carried out the study, analyzed the data, and wrote the paper.

S. Banerjee, D. Devanand, S. Gauthier, R. Howard, K. Lanctôt, C. Lyketsos, E. Peskind, A. Porsteinsson, E. Reich, C. Sampaio, D. Steffens, M. Wortmann, and K. Zhong carried out the study, analyzed the data, and wrote the article.
